# Excellent Degradation Performance of a Versatile Phthalic Acid Esters-Degrading Bacterium and Catalytic Mechanism of Monoalkyl Phthalate Hydrolase

**DOI:** 10.3390/ijms19092803

**Published:** 2018-09-18

**Authors:** Shuanghu Fan, Junhuan Wang, Yanchun Yan, Jiayi Wang, Yang Jia

**Affiliations:** Graduate School of Chinese Academy of Agricultural Sciences, Beijing 100081, China; fanshuanghu@126.com (S.F.); wangjunhuan_1993@163.com (J.W.); 15524115399@163.com (J.W.); 13051534780@163.com (Y.J.)

**Keywords:** degradation, PAEs, hydrolase, molecular dynamics simulation, catalytic mechanism

## Abstract

Despites lots of characterized microorganisms that are capable of degrading phthalic acid esters (PAEs), there are few isolated strains with high activity towards PAEs under a broad range of environmental conditions. In this study, *Gordonia* sp. YC-JH1 had advantages over its counterparts in terms of di(2-ethylhexyl) phthalate (DEHP) degradation performance. It possessed an excellent degradation ability in the range of 20–50 °C, pH 5.0–12.0, or 0–8% NaCl with the optimal degradation condition 40 °C and pH 10.0. Therefore, strain YC-JH1 appeared suitable for bioremediation application at various conditions. Metabolites analysis revealed that DEHP was sequentially hydrolyzed by strain YC-JH1 to mono(2-ethylhexyl) phthalate (MEHP) and phthalic acid (PA). The hydrolase MphG1 from strain YC-JH1 hydrolyzed monoethyl phthalate (MEP), mono-n-butyl phthalate (MBP), mono-n-hexyl phthalate (MHP), and MEHP to PA. According to molecular docking and molecular dynamics simulation between MphG1 and monoalkyl phthalates (MAPs), some key residues were detected, including the catalytic triad (S125-H291-D259) and the residues R126 and F54 potentially binding substrates. The mutation of these residues accounted for the reduced activity. Together, the mechanism of MphG1 catalyzing MAPs was elucidated, and would shed insights into catalytic mechanism of more hydrolases.

## 1. Introduction

At present, the environmental pollution that is caused by human intervention has become a global problem. Phthalic acid esters (PAEs), a group of synthetic compounds, are ubiquitous environmental contaminants. PAEs mainly serve as plasticizers to endow the plastics with properties of flexibility and durability, besides the application as additive in paints, lubricants, adhesives, insecticides, and cosmetics [1]. The molecules of PAEs fail to covalently bind the matrix of plastics, and they are easily released from polymer into various environmental matrices, such as surface water, underground water, landfill leachate, sludge, soil, and sediments [2]. The universal distribution of PAEs in environment exerts great threat on the health of humans and wildlife. Some members of PAEs may act as endocrine disruptors or carcinogen, and particularly lead to reproductive and developmental toxicity, even at low concentration [3,4]. Among PAEs, dimethyl phthalate (DMP), diethyl phthalate (DEP), di-n-butyl phthalate (DBP), di-n-octyl (DOP), butyl benzyl phthalate (BBP), and di(2-ethylhexyl) phthalate (DEHP) have been listed as priority environmental pollutants by United States Environment Protection Agency and China National Environmental Monitoring Centre.

PAEs are refractory organic compounds and resistant to hydrolysis and photolysis in nature. Biodegradation plays a major role in removal of PAEs from environment with the properties of high efficiency, low cost, and environmental safety. The bacteria are dominant among the PAEs-degrading organisms. Multiple bacterial strains with PAEs-eliminating ability have been reported, including the strains from genera *Pseudomonas* [5], *Bacillus* [6], *Arthrobacter* [7,8], *Gordonia* [9], *Rhodococcus* [10], *Sphingomonas* [11,12], and *Mycobacterium* [13]. These strains are distinguished from one another in terms of degradation efficiency towards PAEs and the substrate spectrum. Although some strains can degrade various types of PAEs, such as *Gordonia* sp. JDC-2 [14], *Agromyces* sp. MT-O [15], *Gordonia* sp. Dop5 [16], few strains have been reported to eliminate dicyclohexyl phthalate (DCHP) and BBP with bulky side chains [13,17]. The substrate spectrum to some extent determines or influences degradation performance of strains. There is usually coexistence of multiple types of PAEs in the contaminated environment. Therefore, strains that are capable of degrading various kinds of PAEs have significant potential for bioremediation application. The biodegradation efficiency towards PAEs is affected by diverse environmental factors, such as temperature, pH values, salinity, metal ions, and organic chemicals. Most of the PAEs-degrading strains exhibit the highest degradation efficiency at about 30 °C, pH 7.0–8.0 [5,18], and their tolerance of high concentration of salinity has been rarely reported [19]. Thus, the strains with high degradation efficiency under a broad range of environmental conditions are urgently required for the remediation of PAEs-contaminated environment.

The degradation of PAEs is initiated by cleavage of the two ester bonds, which is regarded as the most critical step throughout the complete degradation process. The dialkyl phthalate hydrolase hydrolyzes the first ester bond of PAEs to produce monoalkyl phthalate, which is catalyzed to PA by monoalkyl phthalate hydrolase. To date, the gene sequences of some dialkyl phthalate hydrolases have been identified, and their hydrolytic activity have been characterized. These hydrolases include M673 [20], EstS1 [21], M11 [22], DphB [23], EstG [24], and EstSP1 [25]. There are only three monoalkyl phthalate hydrolases with the gene sequence available: P8219 MehpH [26], EG-5 MehpH [27], and PatE [28]. All of the three hydrolases are able to catalyze hydrolysis of monoethyl phthalate (MEP), mono-n-butyl phthalate (MBP), mono-n-hexyl phthalate (MHP), and mono(2-ethylhexyl) phthalate (MEHP) to PA, and the P8219 MehpH exceeds the others in catalytic efficiency. Generally, the conserved catalytic triad (Ser-His-Asp/Glu) of α/β hydrolases is involved in the hydrolysis of ester bond, and other amino acid residues play a role in binding substrates. However, for monoalkyl phthalate hydrolases, the mechanism of binding and hydrolyzing monoalkyl phthalates (MAPs) has not been elucidated. Fortunately, molecular docking and molecular dynamics simulation have been adopted to analyze the interaction between the enzyme and corresponding substrates [29,30,31]. These computational methods could be important approaches to investigate combination of monoalkyl phthalate hydrolase and MAPs, detect the key residues, and resolve the catalytic mechanism.

Among the PAEs-degrading strains, *Gordonia* sp. YC-JH1, isolated from petroleum-contaminated soils, possesses the broadest substrate range of PAEs, particularly including the most recalcitrant BBP, DCHP, DEHP, and DOP [32]. According to the results here, the strain YC-JH1 had high degradation efficiency toward PAEs and good adaptability to environment of strong alkaline and high temperature. The pathway of DEHP degradation by strain YC-JH1 was deduced according to the metabolic products. The hydrolase MphG1 was cloned from strain YC-JH1, and heterogeneously expressed in *Escherichia coli*. The hydrolytic activity of recombinant MphG1 towards MAPs was determined. Moreover, the three-dimensional (3-D) structure of MphG1 was modeled. Based on the results of molecular docking and molecular dynamics simulation, the key amino acid residues, putatively involved in combining and catalyzing MAPs, were detected. The function of key residues was confirmed by site-directed mutagenesis. Together, the molecular mechanism of MAPs degradation by hydrolase was proposed.

## 2. Results and Discussion

### 2.1. Excellent DEHP-Degrading Performance of Strain YC-JH1

**Figure 1:**
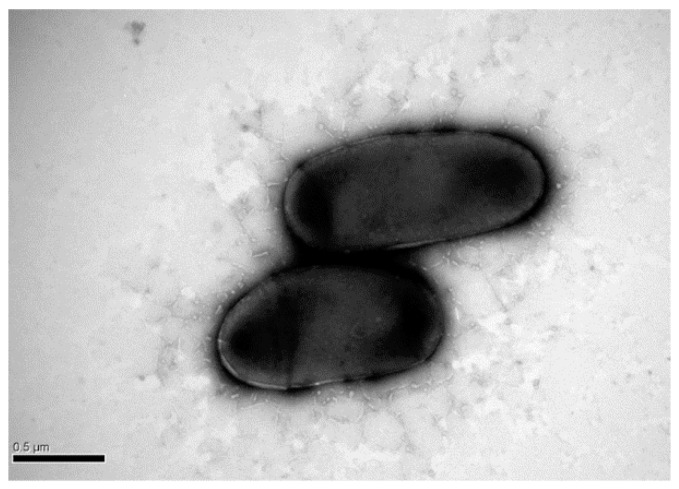
Transmission electronic microscopy (TEM) investigation of *Gordonia* sp. YC-JH1.

**Figure 2:**
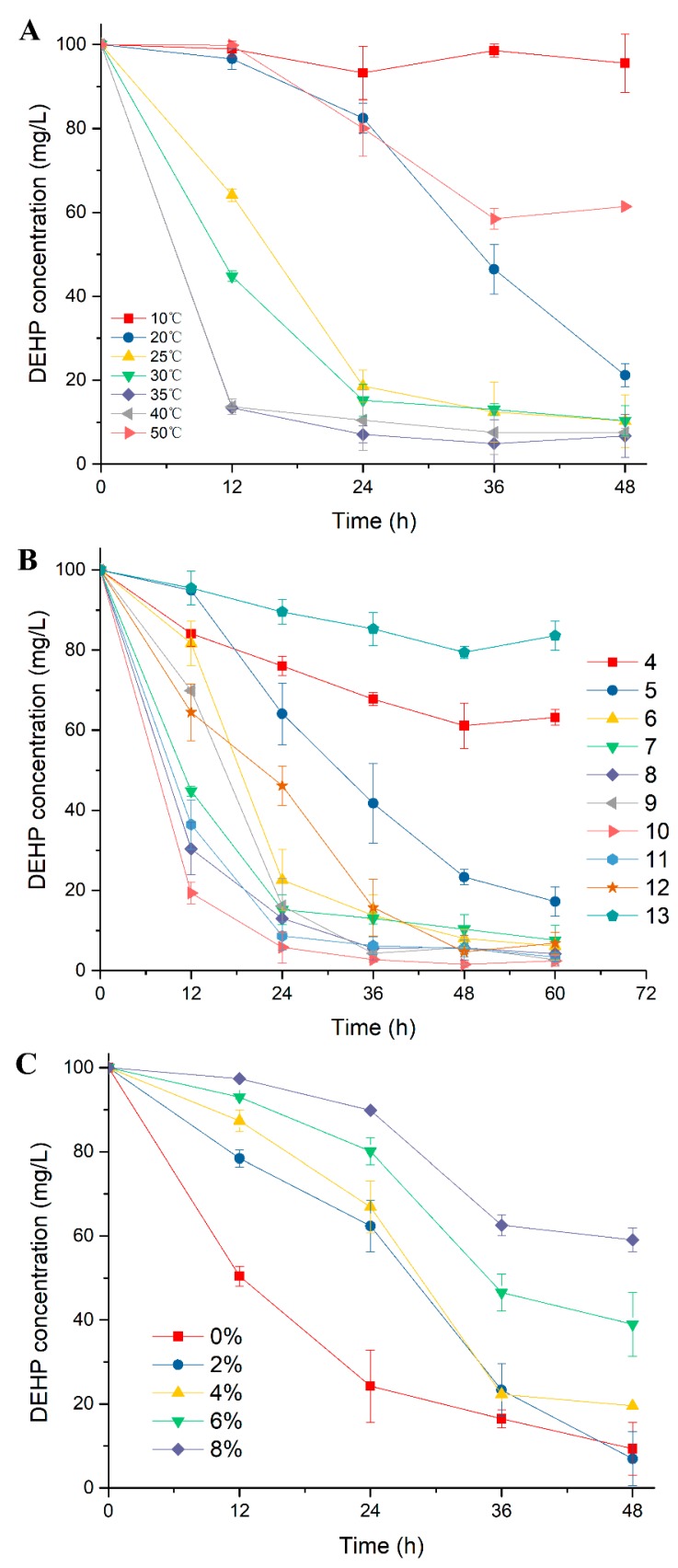
Degradation of di(2-ethylhexyl) phthalate (DEHP) by strain YC-JH1 under a wide range of temperature (**A**), pH (**B**), and concentration of NaCl (**C**).

The strain *Gordonia* sp. YC-JH1, which was capable of efficiently degrading various types of PAEs [32], had no flagella and was (0.4–0.7) × (1.2–1.7) μm in size (Figure 1). Since the degradation performance of strain YC-JH1 might contribute to its application to environment remediation, its capability of eliminating DEHP under various environment conditions was investigated. As shown in Figure 2A, rapid transformation of DEHP by strain YC-JH1 was observed from 25 to 40 °C, with 80%–90% of DEHP being removed within 24 h. The degradation efficiency was the highest at 35 and 40 °C with the similar degradation profiles, and especially approximately 86% of DEHP was transformed after the first 12 h. Therefore, the optimal temperature for DEHP degradation was 40 °C. Most of the PAEs-degrading bacteria had an optimal degradation temperature of 25–30 °C [13,15,19], lower than that of strain YC-JH1. At the optimal temperature, strain YC-JH1 was able to degrade DEHP more effectively than other strains. The strain *Acinetobacter* sp. M673, isolated from contaminated soil of a plastic plant, could degrade 70% of DEHP (100 mg/L) within 12 h [20]. The strain *Mycobacterium* sp. YC-RL4 removed only about 10% of 50 mg/L DEHP within 12 h [13]. It took 24 h to transform about 50% of 100 mg/L DEHP by another phthalate degrader *Agromyces* sp. MT-O [15]. At 25 and 30 °C the degradation ratio of DEHP was comparable to that at 35 and 40 °C after incubation of 48 h, despite of the slightly slower degradation rate during the first 12 h. In addition, the strain YC-JH1 also exhibited good degradation ability at 20 and 50 °C with approximately 80% and 40% of DEHP eliminated within 48 h respectively. This result strongly implied that the enzymes contributing to DEHP degradation were also active at relatively low and high temperature. Together, the strain YC-JH1 possessed high degradation capability towards DEHP over a wide range of temperature. Due to excellent degrading properties, strain YC-JH1 had great potential for environmental remediation under a broad temperature range.

The environmental pH value served important role in DEHP degradation by microorganisms via affecting the activity of causal enzymes. Figure 2B illustrated the profile of DEHP degradation by strain YC-JH1 in TEM medium of initial pH ranging from 4.0 to 13.0. The strain was capable of transforming DEHP effectively under a wide pH range of 5.0–12.0, especially under pH 7.0–11.0. The DEHP degradation was optimal at pH 10.0, where the greatest ratio of degraded DEHP was displayed throughout the experiment period and DEHP was almost completely degraded after 48 h. While most strains preferred a neutral or slightly alkaline environment (pH 7.0–8.0) for phthalate degradation [15,33,34,35,36,37], the alkaline pH of 10.0 was more favorable for strain YC-JH1 to remove DEHP. This strain could degrade DEHP efficiently under strong alkali environment with about 90% of DEHP removed at pH 10.0 and 11.0 after 48 h. Additionally, the degradation process was not completely inhibited under the strong acid (pH 4.0) or alkaline (pH 13.0) environment. These results indicated that strain YC-JH1 was a strong candidate applied to bioremediation under a broad pH range.

The salinity significantly affected xenobiotic metabolism by microbes [38,39]. The influence of salinity on DEHP degradation by strain YC-JH1 was evaluated under a series of concentration of NaCl (0–8%). This strain was capable of removing DEHP effectively under all concentrations, although the decreased removal ratio of DEHP was observed with the increased concentration of NaCl (Figure 2C). While the inhibitory effect arose during the first 24 h under the concentration of 2% and 4%, the degradation rate of DEHP increased during 24–36 h, and at 36 h the percentage of removed DEHP was parallel to the counterpart without NaCl. The concentration of 6% and 8% caused a slower removal ratio of DEHP, but 60% and 40% of DEHP was still removed, respectively, after incubation of 48 h. Thus, this strain could tolerate high salinity and be adopted as bioremediation organism under environment of high salinity [40].

### 2.2. Catabolic Pathway of DEHP by Strain YC-JH1

**Figure 3:**
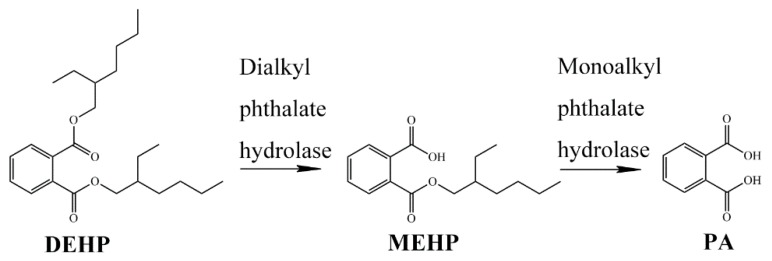
The proposed pathway of DEHP degradation by strain YC-JH1.

Given the complete genome sequence of strain YC-JH1, the putative complete metabolism pathway of PAEs was predicted [32]. PAEs were hydrolyzed to MAPs and PA sequentially by hydrolases. The cleavage of two ester bonds was considered as the most critical steps during PAEs degradation. To confirm the catabolic pathway of DEHP by strain YC-JH1, the intermediates extracted from culture were analyzed by HPLC-MS. From the culture after 0–36 h of incubation, the metabolites were identified as MEHP and PA according to the [M−H]^−^ molecule ion peak (*m*/*z*) of 277 and 165, respectively (Figure S1). The putative catabolic pathway of DEHP was proposed based on the result above (Figure 3). One of the ester bonds of DEHP was hydrolyzed to produce MEHP by dialkyl phthalate hydrolase, and then the remaining single ester bond of MEHP was cleaved by monoalkyl phthalate hydrolase to generate PA. The result conformed to the predicted degradation pathway of PAEs based on the genome sequence, except that the metabolites of PA were not detected by HPLC-MS though tested many times. There were similar intermediates and pathway of PAEs metabolism in *Agromyces* sp. MT-O [15], *Pseudomonas* sp. PKDE1 [41], and *Bacillus megaterium* strain YJB3 [42].

### 2.3. Sequence and Activity Analysis of MphG1

The gene *mphG1* encoded putative monoalkyl phthalate hydrolase of 311 amino acids, 14 more amino acids than EstG2 at the N-terminal [32]. There was no signal peptide and transmembrane domain. The sequence of MphG1 was aligned with other monoalkyl phthalate hydrolases: P8219 hydrolase [26], EG-5 hydrolase [27], and PatE [28] (Figure S2). The pentapeptide Gly-X-Ser-X-Gly (G123-H124-S125-R126-G127) and catalytic triad (S125-H291-D259) conserved in esterase/hydrolase were detected.

**Figure 4:**
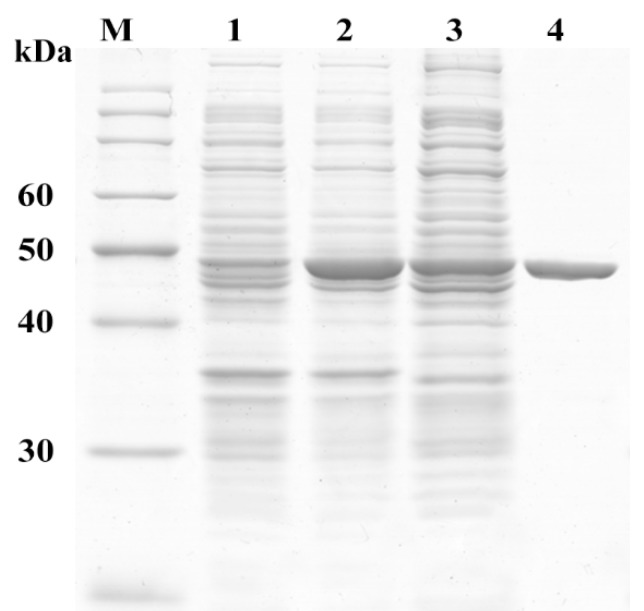
Analysis of expression and purification of recombinant MphG1 by Sodium dodecyl sulfate polyacrylamide gel electrophoresis (SDS-PAGE). Lane M, protein molecular mass marker; lane 1, total protein of ArcticExpress (DE3) harboring pET-*mphG1* without induction; lane 2, total protein of ArcticExpress (DE3) harboring pET-*mphG1* induced by IPTG; lane 3, the supernatant of ArcticExpress (DE3) harboring pET-*mphG1* induced by IPTG; lane 4, purified recombinant MphG1.

To determine the hydrolytic activity of MphG1, the ArcticExpress (DE3) containing vector pET-*mphG1* was induced by 0.1 mM IPTG at 16 °C for 20 h. A great quantity of recombinant MphG1 was expressed and purified to electrophoretic homogeneity (Figure 4). The band of around 50 kDa on the gel was consistent with the calculated molecular weight of recombinant MphG1. The hydrolytic activity of MphG1 was evaluated while using MEP, MBP, MHP, and MEHP as substrate, respectively. As Figures S3–S6 shown, the amount of these MAPs decreased under the action of MphG1. The new peaks appeared with the identical retention time of authentic PA. The result suggested that MphG1 could cleave the ester bond of the MAPs tested to generate PA as product. Among these substrates, MphG1 exhibited the highest activity towards MHP with specific activity of 3.14 U/mg protein (Table 1).

### 2.4. Interaction Mode of MphG1-MAP Complex

The three-dimensional structure of truncated MphG1 was constructed by homology modeling with multiple templates due to the unavailability of the crystal structure of MphG1, and optimized via molecular dynamics (MD) simulation (Figure S7). The stereo chemical quality of the model was validated. As Figure S8 shown, 88.2% of residues were in favored (98%) regions and 95.2% of residues were in allowed (>99.8%) regions, indicating the reliability and favorability of the model of MphG1. The modeled structure contained 10 α-helices, 7 mostly parallel β-strands, and the anti-parallel of β-strand 2. The β-strands were sandwiched between the domain composed of α1 and α10 and the domain composed of α2, α3, α7, and α8.

**Figure 5:**
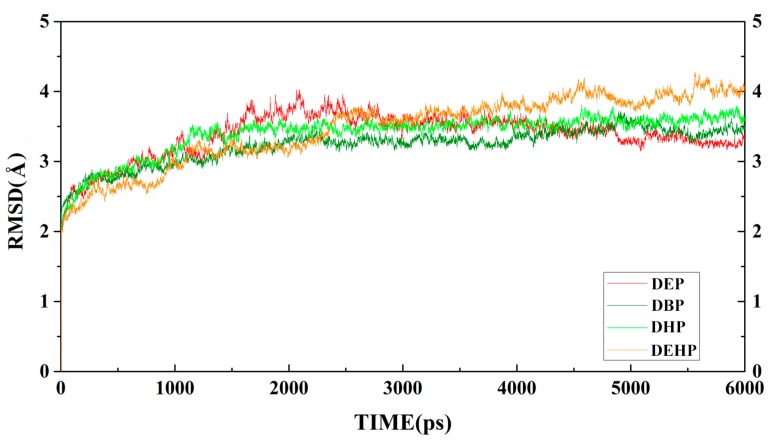
The profile of root mean square deviation (RMSD) values during MD simulation for each MphG1-MAP complex (0–6000 ps).

To investigate the interaction between MphG1 and MAPs, the flexible docking was carried out. After docking, the complexes of each MphG1-MAP with the lowest binding energy were subjected to MD simulation to assess their stability. All complexes reached equilibrium state after 3 ns of MD simulation (Figure 5), and the root mean square deviation (RMSD) values for each MphG1-MAP complex after MD simulation were present (Table 2). The values of binding free energy for the MphG1-MAP complexes were calculated, ranging from −7.86 to −17.34 kcal/mol ( Table 1 and Table S2). Among these, MphG1-MHP had the lowest binding energy, implying the highest affinity between MphG1 and MHP (Table 1). Moreover, the substrate affinity might partly contribute to enzymatic activity, because the specific activity of MphG1 increased with the decreased binding energy of MphG1 and MAPs, except for MEHP (Table 1).

**Figure 6:**
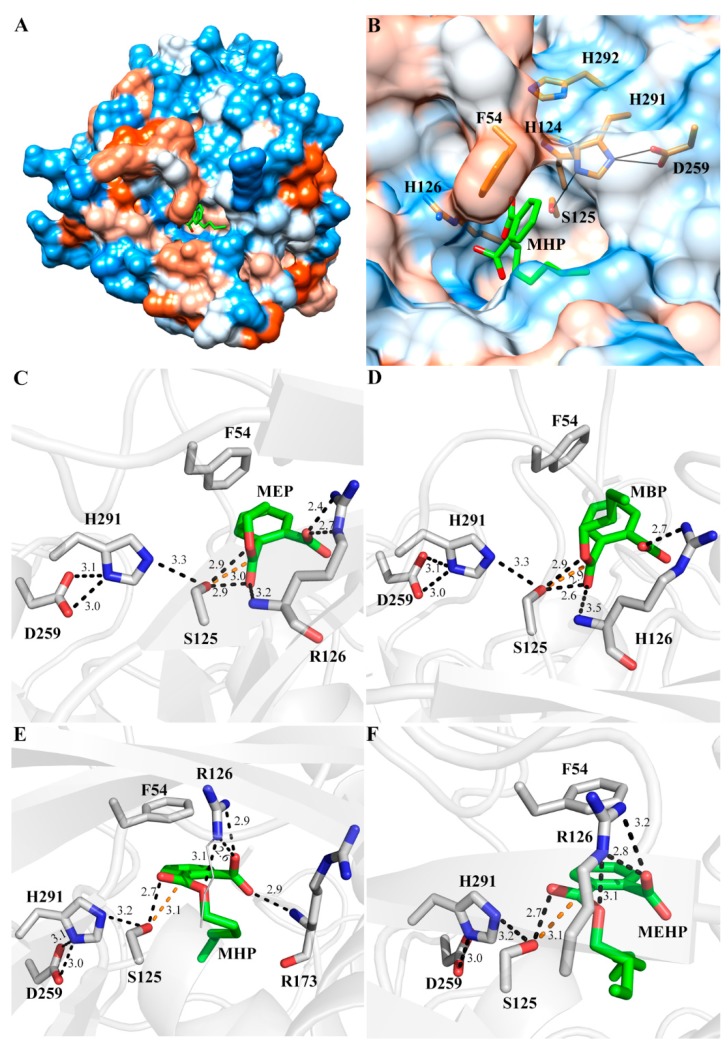
The interaction analysis of MphG1 and MAPs. (**A**) The overall structure of MphG1 binding mono-n-hexyl phthalate (MHP). The hydrophilic regions were displayed in blue and the hydrophobic regions in red. The molecule MHP was shown as green sticks; (**B**) The enlargement of MHP-binding site in (**A**). Some putative key residues around MHP were shown as orange sticks; Detailed structure of MphG1 docking with monoethyl phthalate (MEP) (**C**), mono-n-butyl phthalate (MBP) (**D**), mono-n-hexyl phthalate (MHP) (**E**) and mono(2-ethylhexyl) phthalate (MEHP) (**F**). The MAPs were shown as green sticks, and the amino acids binding and catalyzing MAPs were shown as gray sticks. The distance of hydrogen bond between the residues and MAPs was shown as black dashed lines (Å), and the distance between Oγ of S125 and carbon atom of ester bond of MAPs was shown as orange dashed lines (Å).

According to result of MD simulation, the conserved catalytic triad S125-H291-D259 was located in a hydrophobic cavity (Figure 6A,B), and all of the MAPs were situated in this cavity (Figure 6C–F). Therefore, this cavity might be active site of MphG1, and the catalytic triad would be involved in nucleophilic catalysis against MAPs, a similar case in other α/β-fold hydrolases [41]. The MAPs assumed a similar binding pattern for all complexes (Figure 6C–F). The catalytic triad S125-H291-D259, the key site of hydrolase/esterase family, formed a hydrogen bond network, the similar state that was observed in other hydrolases [43,44]. Additionally, the hydroxyl of S125 formed hydrogen bonds with the oxygen atoms of the ester bond, and would initial nucleophilic attack towards carbon atom of ester bond. The residue F54 hydrophobically interacted with the benzene ring and alkyl chain of MEP and MBP (Figure 6C,D). The benzene ring of F54 was more parallel to the benzene ring of MHP and MEHP, which facilitated formation of π-π stacking, one form of stronger interaction (Figure 6E,F). The positively charged guanidine of R126 electrostatically interacted with the negatively charged carboxyl of MAPs. Besides, the main-chain –NH– group of residue R173 established hydrogen bond with the carboxyl group of MHP (Figure 6E). Based on the results above, the residues S125-H291-D259 were mainly involved in the catalysis of MAPs hydrolysis, and the residues F54, R126, and R173 played an essential role in binding substrates.

### 2.5. The Mutation Analysis and Catalytic Mechanism of MphG1

**Figure 7:**
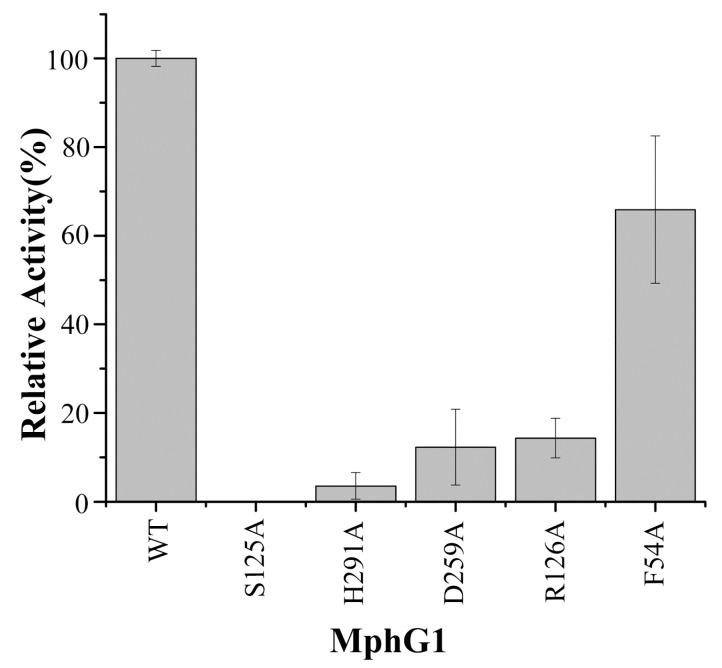
Relative activities of wild type and mutants of MphG1.

**Figure 8:**
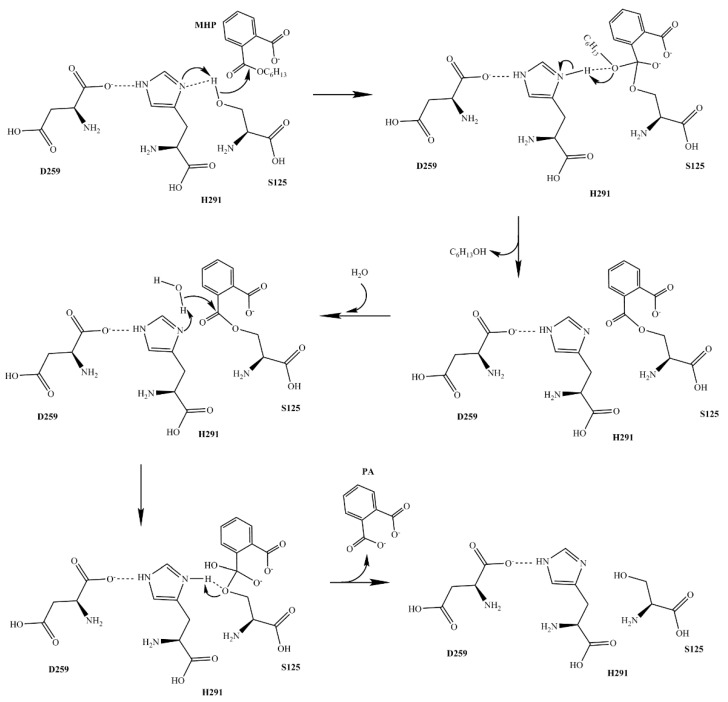
The proposed catalytic mechanism of MphG1.

To confirm the function of putative key amino acids of MphG1, the expression vectors containing one of the mutant codons (S125A, D259A, H291A, R126A, and F54A) were constructed by site-directed mutagenesis, respectively. These target residues were replaced by Ala due to its characteristics of no electric charge, hydrophobicity and less steric hindrance [30]. The corresponding mutants of MphG1 were obtained via heterologous expression and affinity purification (Figure S9). The hydrolytic activity of the wild type and mutants of MphG1 toward MHP was detected. As Figure 7 shown, the S125A mutation abolished the activity of MphG1, and mutant H291A possessed less than 5% of relative activity. The mutant D259A retained slightly higher activity than S125A and H291A. Based on the result here and previous reports [41,45,46], the catalytic mechanism of MphG1 was proposed (Figure 8). The residue H291 acted as general base and deprotonated the hydroxyl of S125, and D259 neutralized the charge of His291. The Oγ of S125 nucleophilically attacked the carbon atom of ester bond of MHP to release alcohol part from MHP, and the PA-MphG1 complex was formed. Afterwards, the carbon atom of PA-MphG1 complex was attacked by the oxygen atom of water molecular, and the S125 returned to initial state and the product PA was released. The other two mutants R126A and F54A displayed 14.33% and 65.89% of activity relative to the wild type MphG1, confirming their important role in binding the carboxyl group and benzene ring of MAPs during the catalytic process. When compared to F54A mutation, the replacement of R126 by Ala resulted in greater decrease in enzymatic activity. Thus, the electrostatic interaction between R126 and carboxyl group of MAPs served more critical role than hydrophobic interaction or π-π stacking between F54 and benzene ring of MAPs.

## 3. Materials and Methods

### 3.1. Chemicals, Medium and Strain

The phthalate esters including DEHP, MEP, MBP, MHP and MEHP were obtained from Dr. Ehrenstorfer GmbH (Augsburg, Germany) or Sigma-Aldrich Co. (Saint Louis, MO, USA). These chemicals were dissolved in methanol with concentration of 20 mg/mL and stored at 4 °C. The solvents of n-hexane, ethyl acetate, and methanol were of HPLC grade from CNW Technologies GmbH (Duesseldorf, Germany). Other chemicals were of analytical grade. The restriction enzymes, ligation enzymes, DNA fragment purification kits and Fast Mutagenesis System for site-directed mutagenesis were obtained from TRANSGEN BIOTECH (Beijing, China). The LB medium and trace element medium (TEM) were prepared as previous report [19]. The binding buffer (10 mM Na_2_HPO_4_·12H_2_O, 10 mM NaH_2_PO_4_·2H_2_O, 500 mM NaCl, 30 mM imidazole, pH 7.4) and elution buffer (10 mM Na_2_HPO_4_·12H_2_O, 10 mM NaH_2_PO_4_·2H_2_O, 500 mM NaCl, 500 mM imidazole, pH 7.4) were applied to the affinity purification of protein. The previously isolated strain *Gordonia* sp. YC-JH1 was adopted as the initial experiment material [32].

### 3.2. DEHP Degradation Assay by Strain YC-JH1

An appropriate amount of the stored strain YC-JH1 was inoculated into 100 mL liquid LB medium in 500 mL Erlenmeyer flask, and incubated at 180 rpm and 30 °C. Once exponential growth phase was reached, the cells were harvested by centrifugation at 5000× *g* for 10 min. The precipitated cells were washed twice by sterile TEM solution, and resuspended in TEM medium to adjust OD_600_ to 0.7. The suspension was employed as inoculum for all of the following investigation with inoculum size of 1.0%.

The strain YC-JH1 suspension was inoculated into 100 mL Erlenmeyer flask containing 20 mL TEM medium supplemented with 100 mg/L DEHP. To determine the DEHP degradation performance of strain YC-JH1, three environmental factors, including temperature (10–50 °C), pH values (4.0–13.0), and NaCl concentration (*weight*/*volume*) (0–8%) were employed, and each batch of experiments was conducted under the substitution of normal condition (30 °C, pH 8.0, 0% NaCl (*weight*/*volume*)) by single environmental factor above. The control without inoculation was conducted under the same condition. All of the culture was incubated at 180 rpm and in dark condition, and the samples were collected at the interval of 12 h for detection of residual DEHP. The culture was vortexed with equal volume of n-hexane, and the organic phase above was subjected to GC-2010 (SHIMADZU, Kyoto, Japan) equipped with ECD detector and RTX-1301 capillary column (30.0 m × 0.25 mm × 0.25 μm). The detection conditions of GC were as follows: nitrogen (purity > 99.999%) as carrier gas (1.0 mL/min), injection volume of 1.0 μL, injection temperature of 300 °C, column temperature of 280 °C, and ECD detector temperature of 300 °C. All of the degradation experiments were independently conducted in triplicate.

### 3.3. Identification of Intermediates of DEHP Degradation

To verify the pathway of DEHP degradation by strain YC-JH1, the metabolites of DEHP were analyzed using HPLC-MS [13]. The prepared inoculum of strain YC-JH1 was inoculated into 400 mL TEM medium (pH 8.0) containing 100 mg/L DEHP, and incubated at 30 °C, 180 rpm. The culture was sampled at the interval of 12 h. The metabolites were extracted by thoroughly mixing the culture with equal volume of ethyl acetate. For each sample, the extractive process was repeated twice and the extractive solution merged together. After the solvent ethyl acetate was evaporated completely by nitrogen stream, the extracts were re-dissolved in 5 mL methanol. The solution of metabolites was filtrated by 0.22 µm filter membrane, and applied to HPLC equipped with a triple quadrupole mass spectrometer (Agilent 6420, Santa Clara, CA, USA) [13]. The solution was directly injected into the mass spectrometer with 10 μL and eluted by 10% water and 90% methanol at a flow rate of 0.2 mL/min. The electro spray ionization (ESI) source and negative ionization mode were adopted. The data from full scan for the mass range of *m*/*z* 50–400 Da was analyzed by Mass Hunter (version A.02.00, Agilent, USA).

### 3.4. Gene Cloning, Expression and Purification of Enzyme

In our previous report, *estG2* was identified as the gene of monoalkyl phthalate hydrolase based on the genome annotation using NCBI Prokaryotic Genome Annotation Pipeline [32]. The ORF was re-predicted in the range of *estG2* plus 100 bp of its upstream and downstream sequence respectively by ORF Finder (available online: https://www.ncbi.nlm.nih.gov/orffinder/) and FGENESB (available online: http://www.softberry.com/berry.phtml?topic=fgenesb&group=programs &subgroup=gfindb), and designated as *mphG1*. The sequence of *mphG1* had deposited in GenBank database under accession number MH674097. The signal peptide and transmembrane domain were predicted by SignalP 4.1 Server and TMHMM Server v. 2.0, respectively. The complete coding sequence of *mphG1* was amplified from the genome of strain YC-JH1 using primers *mphG1*-F and *mphG1*-R (Table S1). The fragment of *mphG1* was cloned into the EcoRI (5′end) and HindIII (3′end) sites of pET32a(+) vector. The resulting vector pET-*mphG1* was transformed into ArcticExpress(DE3) cells. The positive transformant was incubated in LB medium containing 50 μg/mL ampicillin at 37 °C and 180 rpm. Once the OD_600_ of culture reached 0.6, 0.1 mM IPTG was applied to induce the expression of *mphG1* gene at 16 °C. After induction of 20 h, the cells were harvested by centrifugation (5000× *g* for 10 min at 4 °C), resuspended in binding buffer, and disrupted by sonication. The lysate was centrifuged, and the collected supernatant was applied to HisTrap^TM^ HP column, equipped on ÄKTA avant. The column was washed by binding buffer to remove unbound protein, and eluted by elution buffer to collect recombinant MphG1. The protein was dialyzed to remove imidazole and stored in 50 mM Tris-HCl (pH 8.0) with 20% glycerol at −20 °C. The concentration of MphG1 protein was determined by BCA Protein Assay Kit (TIANGEN, Beijing, China).

To yield the mutants of MphG1 (S125A, D259A, H291A, F54A, and R126A), the site-directed mutagenesis was introduced into pET-*mphG1* according to the protocol of Fast Mutagenesis System using primers in Table S1. The PCR products were transformed into ArcticExpress (DE3), and the positive clones were validated by sequencing. The mutants of MphG1 were expressed and purified as the procedure described above.

### 3.5. Hydrolase Activity Assay

To determine the hydrolytic activity of MphG1, the MphG1 was incubated with 0.5 mM MAP in 1 mL of 50 mM Tris-HCl (pH 8.0) at 30 °C for 10 min. The following MAPs were used as substrates, respectively: MEP, MBP, MHP, and MEHP. The reaction was terminated by addition of 100 μL of 1 N HCl. The residual substrates and products were extracted with 1 mL of ethyl acetate. After ethyl acetate evaporating to dryness, the extracts were re-dissolved in 1 mL of methanol for HPLC analysis. The controls without enzyme were carried out under the same condition. The HPLC system (Agilent 1200) with column ZORBAX Eclipase XDB C18 (4.6 mm × 150 mm, 5 μm) was used. For analysis of MEP and MBP hydrolysis, the elution was performed by 60% methanol and 40% water containing 0.1% acetic acid at a flow rate of 0.8 mL/min. For analysis of MHP and MEHP hydrolysis, the elution was performed by 80% methanol and 20% water containing 0.1% acetic acid at a flow rate of 0.8 mL/min. The enzyme activity was measured based on the transformation of MAPs. One unit of enzyme activity was defined as the amount of enzyme required to hydrolyze 1 µmol of MAP per minute under the assay condition. The activity of mutant MphG1 was determined while using the same condition. All of the reactions were performed in independent triplicates.

### 3.6. Molecular Docking and Molecular Dynamics Simulation

To analyze the interaction between MphG1 and the MAPs, the homology modeling of MphG1 was conducted with multiple templates. The sequence of MphG1 was submitted to Modeler9.2, and divided into several regions automatically to search the templates from PDB database. The structures of proteins (1u2e [47], 4lxg [48], 1j1i [49], 4lxh [48], 2pu5 [50], 5jz9 [51], and 5jzb [51]) shared identity of more than 30% with particular region of the 21–311 amino acids sequence of MphG1, and was adopted as templates for homology modeling. The resulted model of 21-311aa of MphG1 via homology modeling was optimized using molecular dynamics (MD) simulation. Then, the structure after MD simulation was validated by Molprobity (available online: http://molprobity.biochem.duke.edu/index.php) [30].

The molecular docking of MphG1 and MAPs (MEP, MBP, MHP, and MEHP) was performed by AutoDock4.0 while using a Lamarkian genetic algorithm. For MphG1 modeling structure, the hydrogen atoms and Kollman charges were added. The spatial structures of MAPs were retrieved from PubChem (available online: http://pubchem.ncbi.nlm.nih.gov/), and the rotations and torsions of MAPs were automatically set in Autodock. A small grid spacing of 0.375 Å was set to allow the search for receptor grid box. The receptor grid box containing the conserved catalytic triad was set at 25 Å × 25 Å × 25 Å with the center point *X* = −18.967, *Y* = 19.958 and *Z* = 8.61. Other docking parameters were set to default. During docking, a maximum number of 100 conformers were considered, and the root-mean-square (RMS) cluster tolerance was set to 2.0 Å. The best docking result of MphG1-MAP complex with lowest binding energy was selected for MD simulation with Amber11. The Amber ff03 force field was applied to MphG1. Na^+^ as counter-ion was added to neutralize the simulation system. The MphG1-MAP complex was surrounded by a cubic periodic box of TIP3P water molecules with a minimum 10 Å distance from every peripheral residue. Electrostatic interaction was evaluated with a cutoff of 10 Å by particle-mesh Ewald (PME) method. Before MD was carried out, 500 steps of steepest-descent minimization and 1500 steps of conjugated gradient minimization were applied to eliminate the unsuitable contact between the atoms. At the beginning of MD, the system was slowly heated from 0 to 300 K during 500 ps in NVT (constant volume and normal temperature) ensemble, and the density equilibrium dynamics was simulated for 500 ps when the main chain of MphG1 was subjected to weak molecular mechanical constraint. The unconstrained equilibration dynamics of the entire system was performed in the NPT (constant normal pressure and normal temperature) ensemble for 6 ns. Throughout the process, the SHAKE algorithm was applied to constrain the hydrogen atom-related covalent bonds.

## 4. Conclusions

The strain *Gordonia* sp. YC-JH1 had broad substrate spectrum, including the most recalcitrant types of PAEs: DEHP, DOP, BBP, and DCHP. This strain exhibited high degradation efficiency towards DEHP under various environment conditions, especially in environment of high temperature, strong alkali or high salinity. Therefore, strain YC-JH1 exceeded its counterparts reported previously in environment adaptability, and could be applied to PAEs removal from contaminated environments. It catalyzed hydrolysis of DEHP to PA as the initial degradation steps. The monoalkyl phthalate hydrolase MphG1 from this strain could adopt MEP, MBP, MHP, and MEHP as substrates. According to the result of molecular docking and MD simulation between MphG1 and MAPs, the catalytic triad S125-H291-D259 was identified and the residues R126 and F54 might be involved in binding substrates. This result was confirmed by the mutation of these key residues. Together, the mechanism of MphG1 binding and catalyzing MAPs was proposed. This result could lay solid foundation for the elucidation of the catalytic mechanism of other hydrolases.

## Figures and Tables

**Table 1:** The binding free energy after molecular dynamics (MD) simulation and specific activity of MphG1 towards monoalkyl phthalates (MAPs).

Substrate	Structure	Binding Energy (kcal/mol)	Specific Activity (U/mg)
MEP	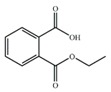	−7.86	2.84 ± 0.14
MBP	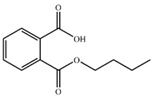	−9.20	2.99 ± 0.04
MHP	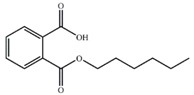	−17.34	3.14 ± 0.06
MEHP	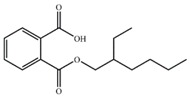	−13.72	2.91 ± 0.09

**Table 2:** The RMSD values for the complexes after MD simulation.

Complex	RMSD/Å
MphG1-MEP	3.3774 ± 0.4
MphG1-MBP	3.2302 ± 0.4
MphG1-MHP	3.4007 ± 0.4
MphG1-MEHP	3.4492 ± 0.3

## Supplementary Materials

The following are available online at http://www.mdpi.com/1422-0067/19/9/2803/s1.
