# Supplementary material for: Excellent Degradation Performance of a Versatile Phthalic Acid Esters-Degrading Bacterium and Catalytic Mechanism of Monoalkyl Phthalate Hydrolase
Source: Int J Mol Sci. 2018 Sep 18;19(9):2803. doi: 10.3390/ijms19092803 (PMC6164851; doi:10.3390/ijms19092803)

Supplementary data

Excellent degradation performance of a versatile PAEs-degrading bacterium and catalytic mechanism of monoalkyl phthalate hydrolase

Shuanghu Fan †, Junhuan Wang †, Yanchun Yan *, Jiayi Wang and Yang Jia

Graduate School of Chinese Academy of Agricultural Sciences, Beijing 100081, China; fanshuanghu@126.com (S.F.); wangjunhuan_1993@163.com (J.W.); 15524115399@163.com (J.W.); 13051534780@163.com (Y.J.)

* Correspondence: yanyanchun@caas.cn; Tel.: +86-10-8210-9685

† These authors contributed equally to this work.

Figure S1. HPLC–MS spectra of DEHP degradation intermediates from the *Gordonia* sp.YC-JH1 culture after incubation of 12 h (A), 24 h (B) and 36 h (C).

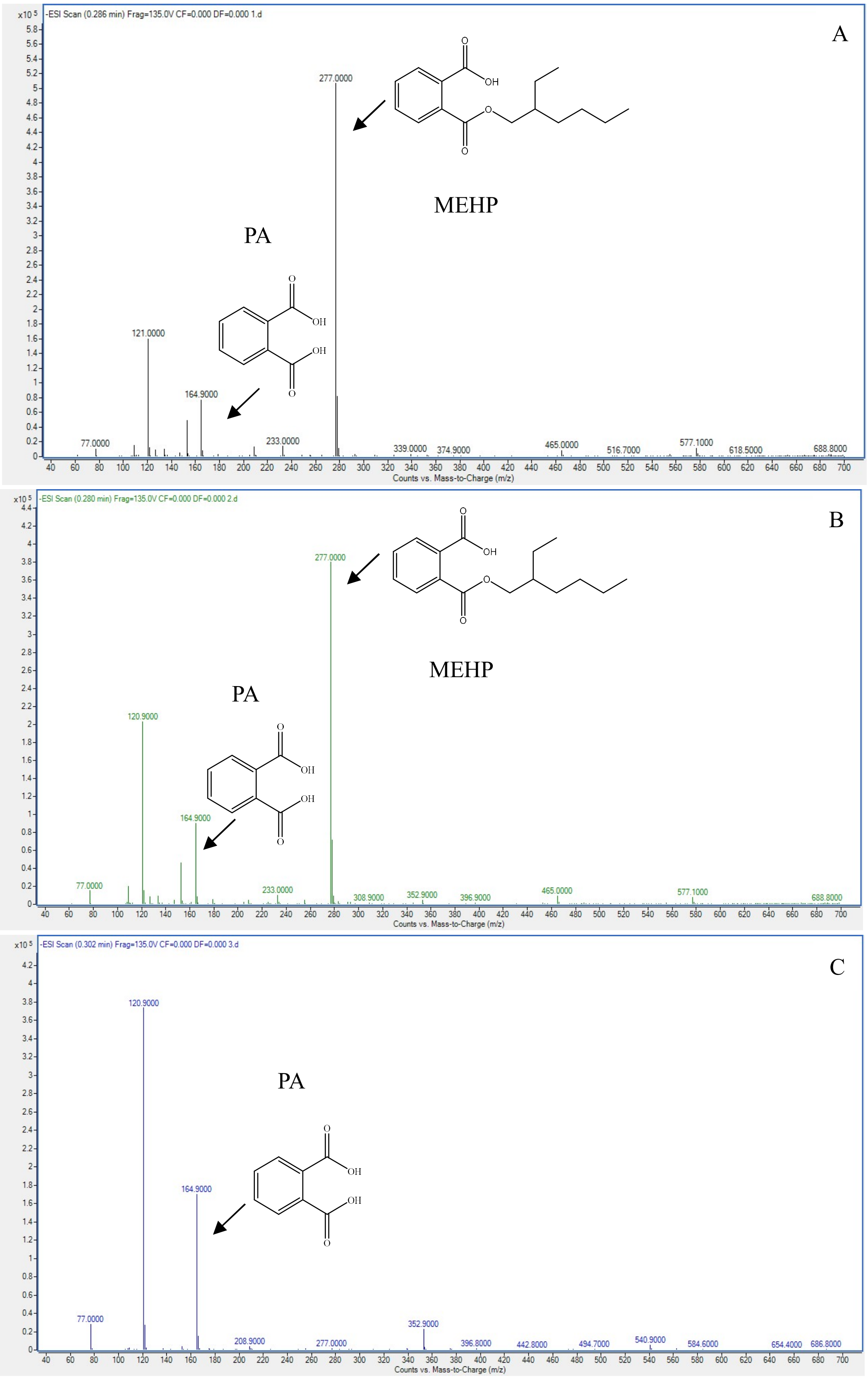

Figure S2. The alignment of MphG1, P8219 hydrolase (BAE78500.1), EG-5 hydrolase (BAU22081.1) and PatE (WP_011599104.1). The conserved Gly-X-Ser-X-Gly motif around the S125 was shown in red rectangle. The catalytic triad S125-H291-259 was indicated by red star.

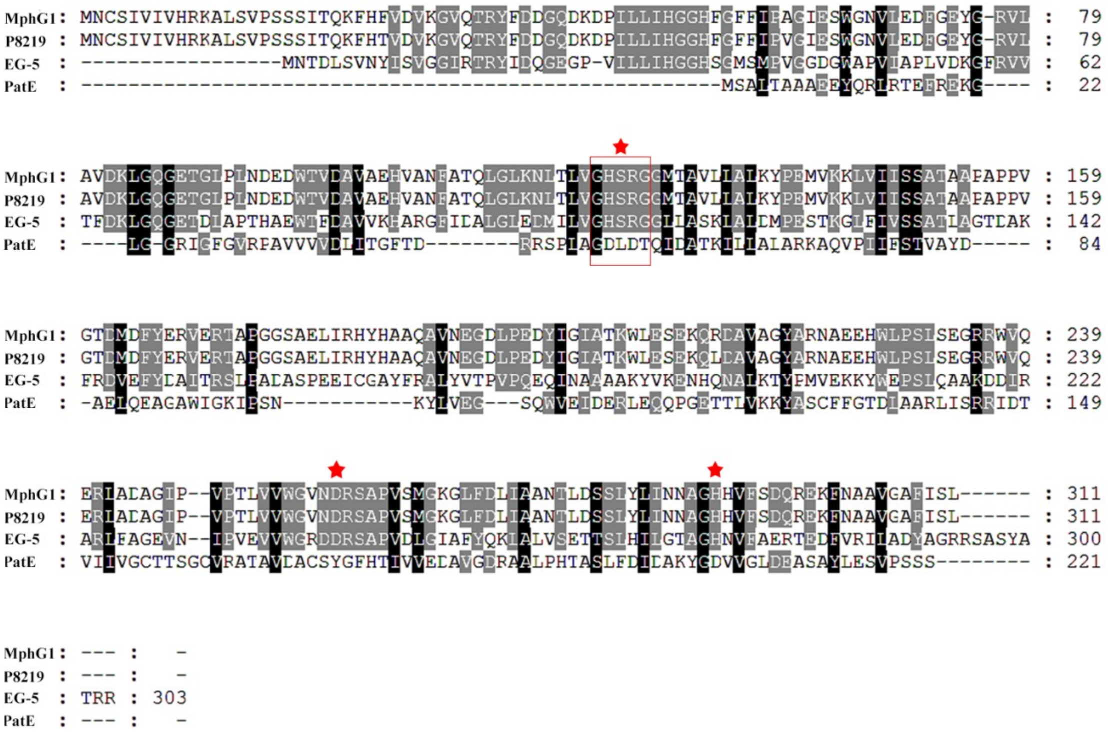

Figure S3. HPLC spectra of MEP hydrolysis by MphG1 (B) compared with control (A).

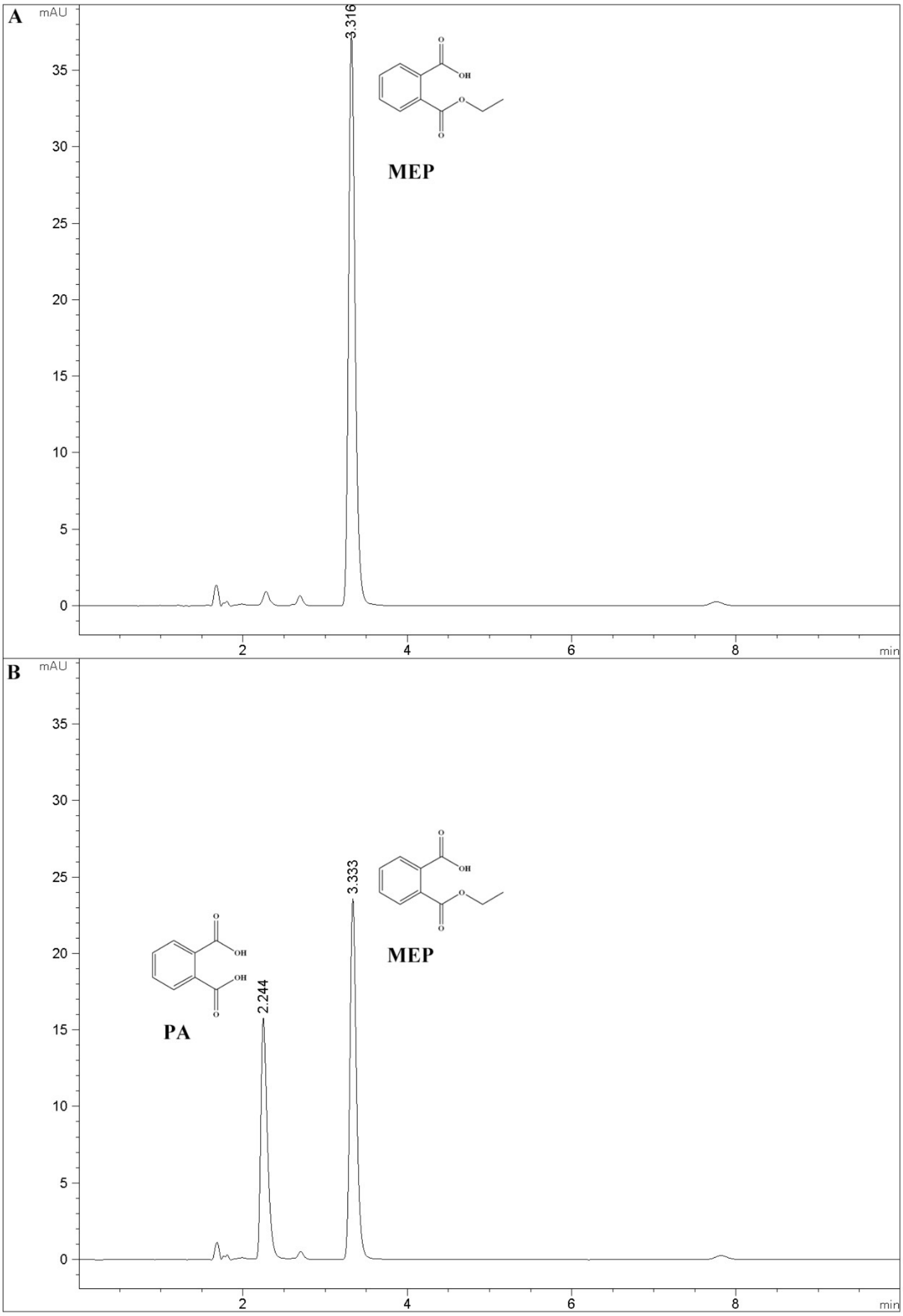

Figure S4. HPLC spectra of MBP hydrolysis by MphG1 (B) compared with control (A).

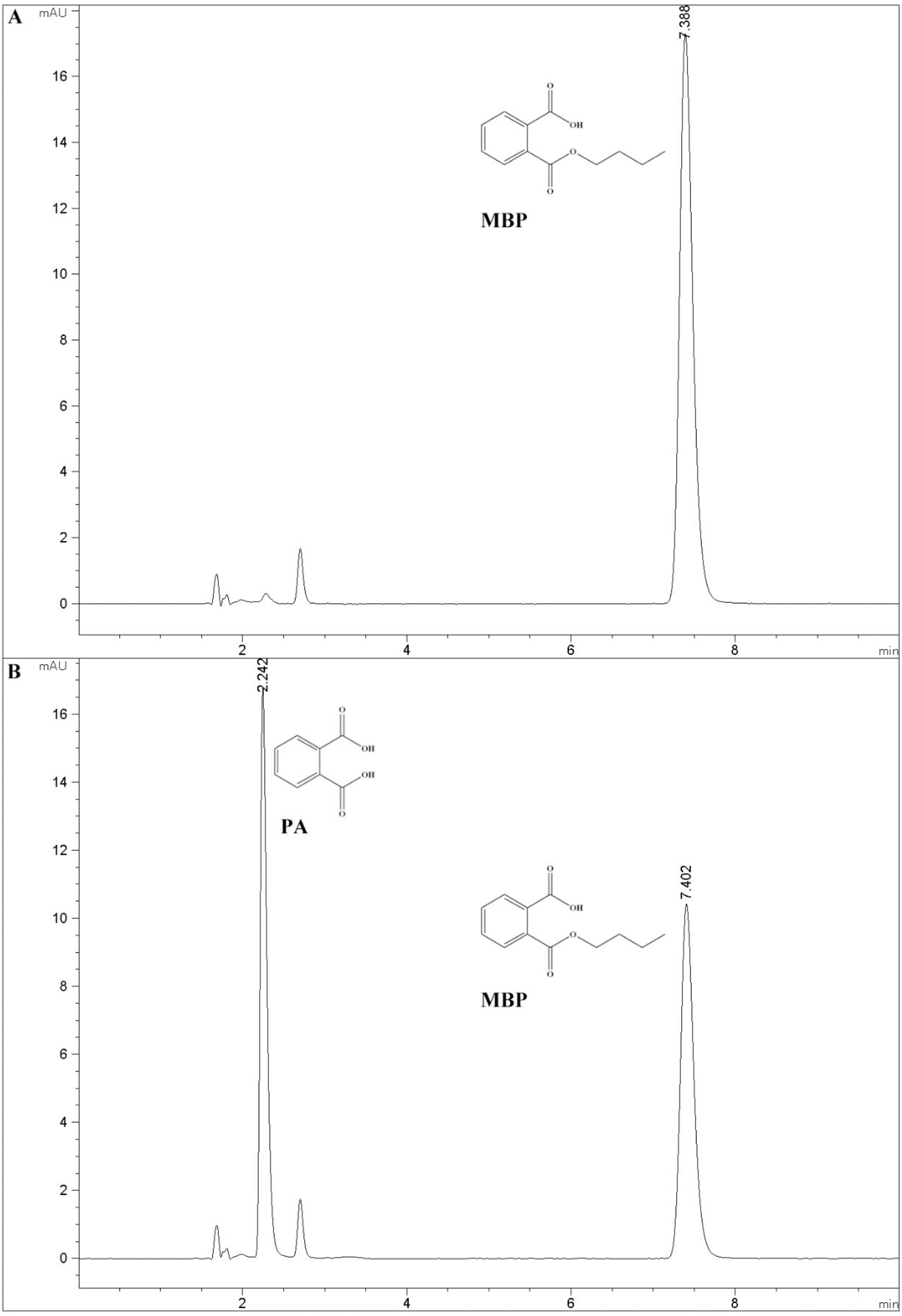

Figure S5. HPLC spectra of MHP hydrolysis by MphG1 (B) compared with control (A).

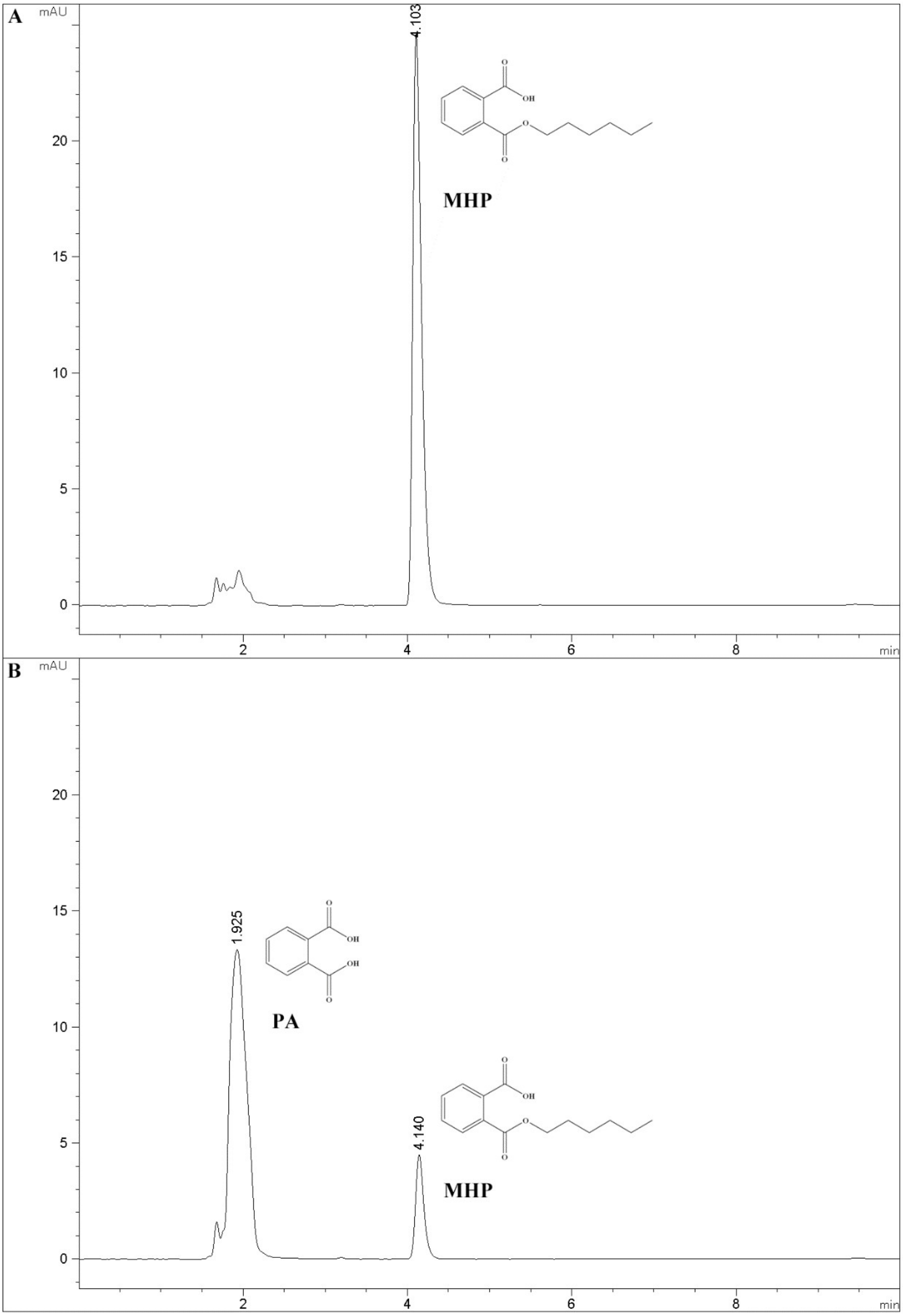

Figure S6. HPLC spectra of MEHP hydrolysis by MphG1 (B) compared with control (A).

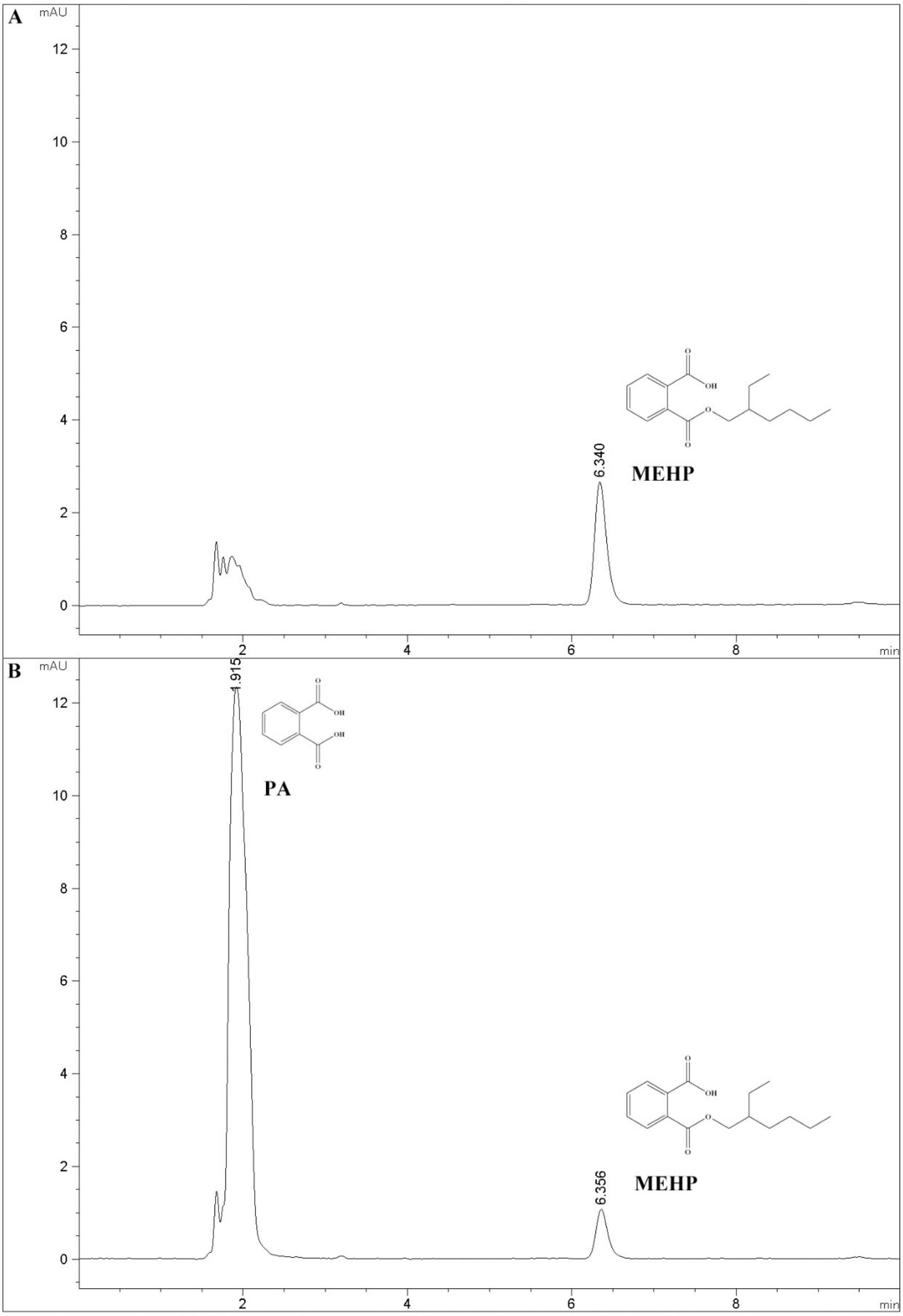

Figure S7. The 3-D structure of MphG1 constructed by homology modeling.

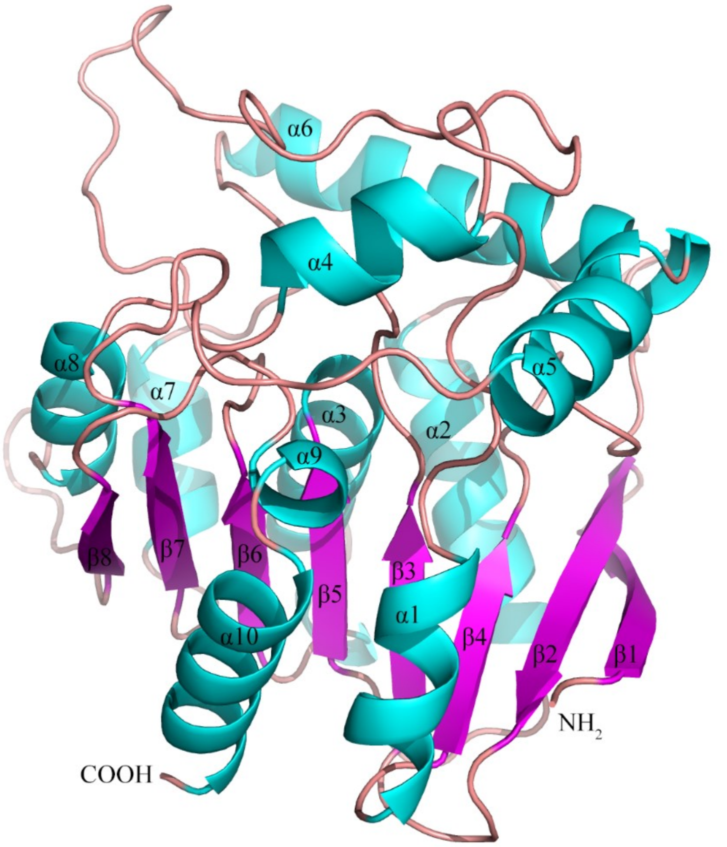

Figure S8. Analysis of purified wild type and mutant MphG1 on SDS-PAGE. Lane M, protein molecular mass marker; lane 1, wild type MphG1; lane 2, S125A mutant; lane 3, H291A mutant; lane 4, D259A mutant; lane 5, H126A mutant; lane 6, F54A mutant.

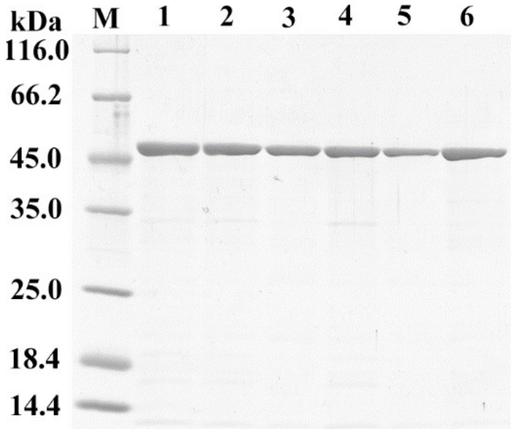

Supplement: Supplementary file 1 [file ijms-19-02803-s001.pdf]
